# Development of the vector education, communication, and training online resource (VECTOR) library: a vector-borne disease education database

**DOI:** 10.1186/s12889-026-27045-5

**Published:** 2026-03-30

**Authors:** Drew T. Lysaker, Emily D. Struckhoff, Indira Chakravarti, Olivia J. Bognanni, Erika T. Machtinger

**Affiliations:** 1 Penn State Extension, The Pennsylvania State University, 201 Old Main, University Park, PA, 16802 USA; 2 The Pennsylvania State University Department of Entomology, 4 Chemical Ecology Laboratory, University Park, PA, 16802 USA

**Keywords:** Cooperative Extension, public health education, one health, vector management, health communication

## Abstract

**Background:**

The emergence of invasive arthropod vectors and the rising prevalence of vector-borne diseases (VBDs) affecting humans and domestic animals have spurred a rapid increase in educational materials. In the United States, the Cooperative Extension System (Extension) can play a role in developing and sharing these resources.

**Main text:**

Extension has historically served rural and agricultural audiences but has expanded to provide information to a variety of other communities throughout the states it serves. One topic that Extension has begun to expand its focus on in certain areas of the U.S. is VBDs. Due to the way each Extension service may focus on and address its own set of issues and topics, regional gaps in material and a lack of information sharing between institutions may arise. We created the Vector Education, Communication, and Training Online Resource (VECTOR) Library as a repository of educational materials related to vectors and VBDs, developed by Extension offices from across the United States, to address issues of knowledge sharing and access. We hope to continue curating VBD content from Extension offices, as well as to add educational materials created by other non-Extension university departments and each state’s state public health department.

**Conclusion:**

This database offers a single platform for accessing educational VBD materials from Extension offices nationwide.

## Background

Arthropod vectors, such as ticks and mosquitoes, and vector-borne diseases (VBDs), including dengue, West Nile virus, Lyme disease, Eastern equine encephalitis, and Chagas disease, pose a significant threat to human and animal health worldwide. In the United States, VBDs have become an increasing concern as the ranges of native vector species expand, new species of vectors or vector-borne pathogens introduced, and disease prevalence rises [1–3]. In response, a growing number of institutions and organizations are developing and implementing educational initiatives aimed at improving public awareness and preventing VBDs.

Among these organizations are the land-grant university system institutions, which through their Cooperative Extension (Extension) offices have long served as trusted sources of science-based information for rural and agricultural communities [4, 5]. Extension bridges the gap between scientific research and the public by providing accessible educational materials and programming [6]. Each U.S. state and territory maintains its own land-grant universities and Extension services to address local needs [7]. In recent decades, Extension services in certain states and locations have increasingly expanded their focus to include vectors, VBDs, and vector management; however, gaps remain in topic coverage and the distribution of related educational materials across regions [5, 6, 8–10].

Extension educators are expected to provide information on a broad range of topics, with some topics, such as VBDs, potentially falling outside their primary area of expertise or specialization. Although several institutions have begun to develop specialized programs and personnel focused on vectors and VBDs, this expertise is not yet widespread [10–12]. In addition to these potential gaps in educator specialization, Extension resources such as fact sheets and infographics have traditionally been maintained on individual university or Extension websites and are not consistently shared across institutions or states. As the role of Extension expands to address human health and engage broader audiences, there is a growing need for a centralized, accessible repository of educational materials encompassing vectors and VBDs that impact humans and their associated animals.

We identified only two other online databases that compile Extension materials nationally, neither of which focuses specifically on vectors or vector-borne diseases (VBDs) [13–15]. VetPestX is an online database containing pesticide label information and related resources, and this resource is housed on the *Veterinary Entomology* website, which also includes expert directories and materials addressing animal pests [13, 14]. In addition, the Extension Foundation hosts a website tool that allows users to search Extension webpages through a custom search engine [15]. However, both tools are limited in scope, as neither provides comprehensive coverage of vector or VBD content, and the custom search engine did not consistently retrieve relevant materials on these topics during use in October 2025.

Given these limitations and the variability in Extension educator expertise, we recognized a need to consolidate existing Extension materials on VBDs into a single, accessible platform. To address this need, we created the Vector Education, Communication, and Training Online Resource (VECTOR) Library. This open-access database compiles educational materials related to vectors and VBDs developed by Extension offices at institutions across the United States [16]. The VECTOR Library enables Extension educators, public health professionals, and other stakeholders to locate and share science-based VBD resources across institutional and state boundaries. Ultimately, we hope this reduces the burden of finding reliable information, increases the dissemination of resources, and decreases the amount of time it takes for those seeking information about vectors and VBDs to locate relevant information.

## Construction and content

We identified taxonomic groups of vectors that are currently considered or were historically important biological vectors of both endemic and introduced pathogens or may be considered nuisances to humans, companion animals, and livestock in the United States. These groups included ticks (Ixodida), mosquitoes (Diptera), keds (Diptera), sand flies (Diptera), biting midges (Diptera), kissing bugs (Hemiptera), fleas (Siphonaptera), and lice (Psocodea).

To find educational materials containing content regarding these taxonomic groups, we used search terms from the vector groups we defined above: tick(s), mosquito(es), kissing bug(s), flea(s), louse, lice, ked(s), sand fly, sand flies, and biting midge(s). We searched the websites of each extension institution in all fifty U.S. states and five U.S. territories, including American Samoa, Guam, the Commonwealth of the Northern Mariana Islands, Puerto Rico, and the U.S. Virgin Islands, using the identified search terms. Web searches using the institution’s name and the search terms defined above were also conducted to locate relevant materials. We began searching each institution’s primary Extension site on June 4, 2024, and ended our initial search for Extension materials on April 14, 2025.

As our initial focus for the database was Extension materials related to vectors and VBDs, we decided that materials must be published or created by the Extension at a given institution and contain substantial vector or VBD content related to our predefined groups to be included. To meet the publication requirement, each resource had to include an official note or visible branding indicating authorship by Extension. We considered publications to contain a significant amount of content if they included at least one paragraph or section devoted to at least one of the arthropod vector groups or to at least one VBD. The primary reasons for these two requirements were to ensure that included products had undergone some internal review process and to limit the scope of the database to only vectors and VBDs, as opposed to all nuisance and pest arthropods.

For materials about specific vector species, we included the common name of the species if they were found on the Entomological Society of America’s (ESA) Common Names of Insects Database [17]. We followed the ESA common names of species with the Latin binomial in parentheses. If no ESA common name existed, we listed only the species’ Latin binomial. We also created a subcategory within the species selection for articles that do not focus on a specific species but instead cover a larger group as a whole or contain only limited information about multiple species. Once we collected and sorted materials by vector groups or species, we further categorized them to support filtering and search functionality (Table 1).

**Table 1:** Descriptions of the categories users can utilize to filter results within the VECTOR Library database

Category	Subcategory/Description^a^
State	Each U.S. state, district, and territory represented in the library with vector or vector-borne disease materials.
Category	Media through which products are created and distributed.
Language	Languages in which materials are written or spoken.
Cost	Indicates whether a material is free or not.
Focus	Thematic content emphasis of the resource (e.g., human-centric, livestock-focused, companion animal-focused).
Vector Group	Arthropod groups biologically transmitting disease-causing pathogens (e.g., ticks, mosquitoes, fleas).
Species	Species common name, if included in Entomological Society of America Common Names of Insects Database, followed by the Latin binomial in parentheses.
Audience	Target groups based on needs and roles (e.g., general public, veterinarians, Extension professionals, public health practitioners).
Year Created/Reviewed	Date materials were last created, updated, or reviewed (range: 1904–2025).
Updated within 3 years of 2025	Binary filter showing whether a resource was updated or published between 2022 and 2025.
CDC Geographic Region	Division of U.S. states into regions defined by the U.S. Centers for Disease Control and Prevention^b^

^a^ For more information about each category and the different subcategories, please visit the category definitions page (https://www.vector-education.org/category-definitions.

^b^ The CDC geographic regions are defined on the CDC National Center for Health Statistics Geographic division or region page (https://www.cdc.gov/nchs/hus/sources-definitions/geographic-region.htm)

Although materials were not reviewed for content accuracy, we assigned two team members from the VectorED Network to conduct an initial review and quality control of materials identified by the project team, ensuring that only materials meeting the specified criteria were included. They also reviewed each product to ensure that the assigned categories adhered to the definitions outlined. Our quality control team ensured that links were functioning, converted the titles of materials to American Psychological Association 7th Edition title case [18], verified that other applicable categories for sorting were correct, and ensured that common names and Latin binomials of all listed species were spelled correctly and followed the ESA common names if appropriate. Duplicate or recurring materials, such as annual updates or surveillance reports, were identified by the reviewers, and only the most recent version was included in the database. For materials published by multiple counties in the same Extension service that were duplications or contained only minor changes to grammar and structure without updates, we listed the original version of the material. Reviewers were responsible for checking for materials that were considered duplicates or contained only minor changes.

## Utility and discussion

### Current materials

**Fig. 1:**
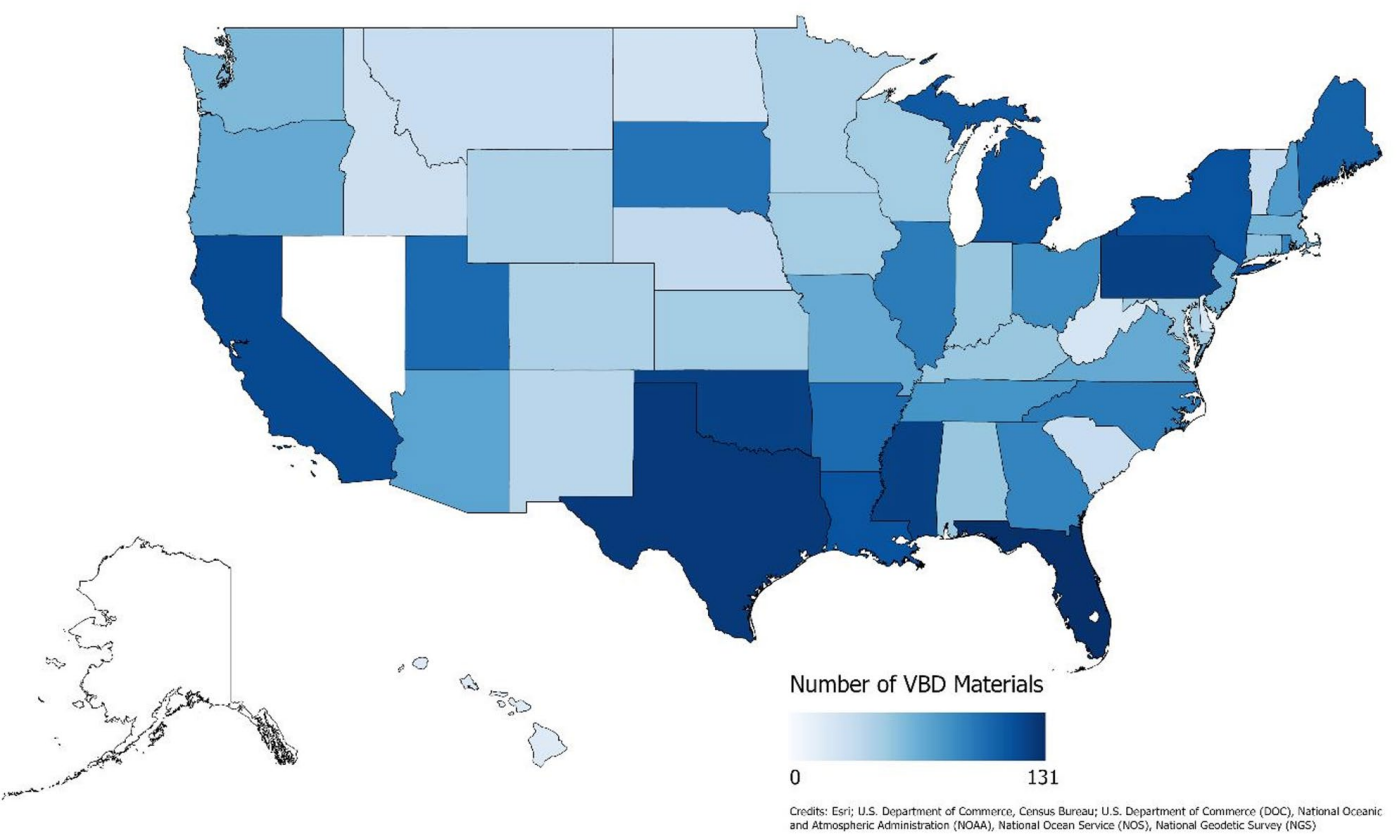
The number of vector-borne disease educational materials in the VECTOR Library as of October 2025. Darker shaded states have a higher number of educational materials. Alaska and Hawaii are not to scale

We launched the VECTOR Library in April 2025, initially containing 1,369 products across 17 categories, including website articles, fact sheets, short videos, and recorded webinars. In May 2025, we completed an update to the VECTOR Library, bringing the total number of products to 1,408. We also began advertising the database at this time. In October 2025, we made another update to the database to refine the materials included, in accordance with our inclusion criteria as described above, which brought the total number of materials to 1,406. The earliest Extension publication related to an arthropod vector or vector-borne disease that we found uploaded online was originally published in 1904, while the most recent materials meeting our criteria were published in 2025. As of the most recent update in October 2025, only two states had no identifiable Extension materials containing substantial content related to vectors or VBD at the time of this publication (Fig. 1). In contrast, the state with the greatest output, Florida, had 131 Extension-produced materials, published either independently or in collaboration with another state’s Extension program (Fig. 1). Of the U.S. territories, Puerto Rico and American Samoa had 2 products and 1 product, respectively, related to the vector groups we included. We were unable to find materials from Extension offices for institutions in Guam, the U.S. Virgin Islands, and the Northern Mariana Islands.

### User interface and visitations

The VECTOR Library is an open-access resource that can be accessed by anyone with a web browser [16]. To aid in finding specific materials, users can filter materials using a series of category filters (Table 1). All materials included in the database are linked back to the Extension website or channel that hosted the material or to a PDF link if we could not find a link to the material on an institutional website. From its launch in April to Nov 30th, 2025, a total of 2,594 unique users visited the VECTOR Library website.

Materials may also be added to the VECTOR Library through user submissions via an online form. Once a user has completed a submission, VECTOR Library team members are notified, and each entry is assigned to a reviewer to verify that it meets the established inclusion criteria before being added to the website. Between the Library’s launch in April and September 1, 2025, four submissions were received from state and county public health organizations.

### Continued curation

Our team has established protocols to guide curation and maintenance of VECTOR Library materials, with updates scheduled annually. These updates include verifying the functionality of existing links, replacing or correcting any links that may no longer function, and identifying newly published materials to be included in the next version of the database. Version history and updates can be viewed on the citations page, located on the site.

### Future additions

Going forward, our goal is to expand the VECTOR Library to also include resources on vectors and VBDs developed by non-Extension University departments and state public health entities. Once these additional materials are curated, we will introduce a new filtering category, allowing users to sort resources by the type of institution that produced them. We also hope to add a new filtering category with the release of public health materials that allow users to filter materials by whether they contain a significant amount of information related to a specific VBD.

## Conclusions

The continued emergence and expansion of VBDs in the United States highlights an urgent need for publicly available, science-based educational materials to support prevention and management. The development of the VECTOR Library represents a significant step toward addressing this challenge by consolidating science-based Extension-produced educational resources from across the nation. The VECTOR Library also improves discoverability, promotes consistency in science-based messaging, and fosters collaboration among Extension educators, public health practitioners, and academic partners.

Looking ahead, continued curation and planned expansion of the VECTOR Library to include materials from non-Extension University departments and state public health entities will strengthen its role as a comprehensive, national hub for vector and VBD education. It will also ensure that the library is relevant for use by a wide range of target audiences, including extension educators, public health professionals, university employees, pest management professionals, and the general public. Ultimately, the VECTOR Library serves as a model for integrating, organizing, and disseminating education and training resources that enhance public understanding of vectors and VBD prevention and management.

## Data Availability

The contents of the VECTOR Library are available at https://www.vector-education.org/. Anyone with access to a web browser can access the VECTOR Library and any materials linked inside the database.

## Abbreviations

CDC: Centers for disease control and prevention
VBD: Vector-borne disease
VECTOR Library: Vector education, communication, and training online resource library
